# An Electrochemical Immunosensor Based on Carboxylated Graphene/SPCE for IgG-SARS-CoV-2 Nucleocapsid Determination

**DOI:** 10.3390/bios12121161

**Published:** 2022-12-13

**Authors:** Luciana de Souza Freire, Camila Macena Ruzo, Bárbara Batista Salgado, Ariamna María Dip Gandarilla, Yonny Romaguera-Barcelay, Ana P. M. Tavares, Maria Goreti Ferreira Sales, Isabelle Cordeiro, Jaila Dias Borges Lalwani, Robert Matos, Henrique Fonseca Filho, Spartaco Astolfi-Filho, Ştefan Ţălu, Pritesh Lalwani, Walter Ricardo Brito

**Affiliations:** 1 Department of Chemistry, Institute of Exact Sciences, Federal University of Amazonas, Manaus 69067-005, AM, Brazil; 2 Instituto Leônidas e Maria Deane (ILMD), Fiocruz Amazônia, Manaus 69067-005, AM, Brazil; 3 BioMark@UC, Department of Chemical Engineering, Faculty of Sciences and Technology, University of Coimbra, 3030-790 Coimbra, Portugal; 4 Department of Physiological Sciences, Institute of Biological Sciences, Federal University of Amazonas, Manaus 69067-005, AM, Brazil; 5 Faculty of Pharmaceutical Sciences, Federal University of Amazonas, Manaus 69067-005, AM, Brazil; 6 Amazonian Materials Group, Federal University of Amapá (UNIFAP), Macapá 49100-000, AP, Brazil; 7 Laboratory of Nanomaterials Synthesis and Nanoscopy (LSNN), Federal University of Amazonas (UFAM), Manaus 69067-005, AM, Brazil; 8 PPGBIOTEC, Federal University of Amazonas, Manaus 69067-005, AM, Brazil; 9 The Directorate of Research, Development and Innovation Management (DMCDI), The Technical University of Cluj-Napoca, Constantin Daicoviciu Street, No. 15, 400020 Cluj-Napoca, Romania

**Keywords:** immunosensor, SARS-CoV-2, N protein

## Abstract

The COVID-19 pandemic has emphasized the importance and urgent need for rapid and accurate diagnostic tests for detecting and screening this infection. Our proposal was to develop a biosensor based on an ELISA immunoassay for monitoring antibodies against SARS-CoV-2 in human serum samples. The nucleocapsid protein (N protein) from SARS-CoV-2 was employed as a specific receptor for the detection of SARS-CoV-2 nucleocapsid immunoglobulin G. N protein was immobilized on the surface of a screen-printed carbon electrode (SPCE) modified with carboxylated graphene (CG). The percentage of IgG-SARS-CoV-2 nucleocapsid present was quantified using a secondary antibody labeled with horseradish peroxidase (HRP) (anti-IgG-HRP) catalyzed using 3,3′,5,5′-tetramethylbenzidine (TMB) mediator by chronoamperometry. A linear response was obtained in the range of 1:1000–1:200 *v/v* in phosphate buffer solution (PBS), and the detection limit calculated was 1:4947 *v*/*v*. The chronoamperometric method showed electrical signals directly proportional to antibody concentrations due to antigen-antibody (Ag-Ab) specific and stable binding reaction.

## 1. Introduction

The coronavirus disease 2019 (COVID-19) began in late 2019, and it has affected billions of people worldwide [1]. Severe acute respiratory syndrome coronavirus 2 (SARS-CoV-2) is the third species of betacoronavirus to cause outbreaks in recent decades and the first species to generate a high number of infections and deaths resulting from the effects of the disease worldwide [2].

SARS-CoV-2 has spike (S), membrane (M), envelope (E), and nucleocapsid (N) proteins in its structure. The N protein is the most abundant protein in the coronavirus. It is a highly conserved immunogenic phosphoprotein that is involved in modulating cell signaling pathways and viral genome replication, and therefore, has the potential to be used as a specific biomarker for the diagnosis of COVID-19 [3]. Consequently, it has the potential to enable specific detection of lgM/lgG antibodies [4].

For the detection of SARS-CoV-2, there are conventional testing methods such as reverse transcription polymerase chain reaction (RT-PCR), which is considered to be the gold standard for SARS-CoV-2 detection; enzyme-linked immunosorbent assay (ELISA); chemiluminescent immunoassay (CLIA); or through rapid tests [5]. These conventional tests have limitations, such as high cost, time-consuming, the need for a laboratory structure for their realization, and the demand for an immunological window [6]. However, electrochemical techniques have many advantages, such as allowing fast and economical analysis, low detection limit, practicality, and allowing on-site analysis [7,8,9].

Considered to be an excellent tool for diagnostics, a screen-printed carbon electrode (SPCE) provides lab to market capability for many biosensors [10,11,12]. This electrode can also provide an inexpensive, disposable kit for detecting in a simple and fast way, in addition to quantitatively analyzing biomolecules in a sample matrix [13,14,15]. Different matrices of materials can be applied to improve the performance characteristics of a biosensor and to immobilize biomolecules on the electrode surfaces [13].

Graphene is a monolayer of carbon atoms packed into a two-dimensional (2D) honeycomb-shaped lattice and it is a basic building block for graphitic materials of all other dimensions. Due to its unique electrical, thermal, optical, mechanical, and biological properties, graphene has attracted a lot of attention. This material exhibits an excellent ability to promote electron transfer for some enzymes and catalytic behavior towards small biomolecules, making graphene extremely attractive for biosensors [16]. Graphene carboxylation is the simplest way to introduce functional groups on a graphene surface. Carboxyl groups have been used as a binding site for the immobilization or conjugation of peptides and proteins, enzymes, antibodies, targeting agents, and natural or synthetic amine-containing polymers [17,18,19,20,21,22,23].

In the present work, we designed an unlabeled electrochemical biosensor with N protein as a specific receptor to detect the IgG-SARS-CoV-2 antibody. N protein was immobilized on the SPCE that was modified with carboxylated graphene (CG) to be a selective and sensitive analysis tool. Cyclic voltammetry (CV) and electrochemical impedance spectroscopy (EIS) were used to investigate the proposed biosensor using [Fe(CN)_6_]^4−/3−^ as a redox probe. Reagent-free amperometric detection was prepared by trapping a TMB (3,3’,5,5’-tetramethylbenzidine) redox mediator on the surface of a CG-modified SPCE. TMB is a redox mediator that provides a fast response and analytical sensitivity [24,25,26,27,28,29,30,31,32,33,34]. The immunosensor has good sensitivity, reproducibility, and stability. No electrochemical biosensor has been reported for the detection of IgG-specific viral antibodies based on N protein with CG-modified SPCE in real human serum samples.

## 2. Materials and Methods

### 2.1. Reagents and Samples

Graphene nanoplatelets (XG sciences, Lansing, MI, USA), sulfuric acid (H_2_SO_4_), nitric acid (H_2_NO_3_), disodium hydrogen phosphate (Na_2_HPO_4_), monosodium dihydrogen phosphate (NaH_2_PO_4_), 1-ethyl-3-[3-(dimethylamino)propyl]-carbodiimide (EDC), N-hydroxysuccinimide ester (NHS), and bovine serum albumin (BSA) were purchased from Sigma-Aldrich and Merck Co. Inc. (Rahway, NJ, USA) and used without further purification. N protein was synthesized and purified at the Federal University of Amazonas [35]. 1-Step™ Ultra TMB-ELISA substrate was acquired from Thermo Fisher™ Scientific (Massachusetts, New York, NY, USA). Peroxidase affinity purified goat anti-human IgG (H + L) antibody coupled with peroxidase enzyme KPL (Gaithersburg, MD, USA) (KPL0.1 mol L^−1^ PBS of pH 7) was prepared using Na_2_HPO_4_, NaH_2_PO_4_, and Milli-Q water. In addition, a solution containing 5 mmol L^−1^ K_3_Fe(CN)_6_/K_4_Fe(CN)_6_ with a ratio of 1:1 prepared in 0.1 mol L^−1^ KCl was used as a redox probe in the electrochemical measurements. All the electrochemical experiments were performed at room temperature (22 ± 0.5 °C) and without stirring.

Due to the use of the N protein as an IgG-specific antigen, the proposed method allowed us to detect IgG in biological fluids without interfering with other biomolecules in real samples. The Federal University of Amazonas provided the human blood serum samples. This study was approved by The Research Ethics Committee of the Federal University of Amazonas (CAAE:34906920.4.0000.5020) following Brazilian law and the Declaration of Helsinki.

### 2.2. Apparatus and Procedures

The electrochemical characterization was performed by cyclic voltammetry (CV), electrochemical impedance spectroscopy (EIS), and chronoamperometry (CA) using an Autolab PGSTAT204 potentiostat/galvanostat equipped with the FRA32M EIS module and NOVA software, 2.1.4 version (Metrohm Autolab, Utrecht, The Netherlands).

The disposable SPCEs (reference 110) were acquired from Metrohm Dropsens (Netherlands) with a three-electrode configuration, formed by using two carbon electrodes, i.e., working (WE) and counter (CE) electrodes, and a reference pseudo-reference electrode (RE) with Ag/AgCl. CV was performed in a potential range between − 0.6 and 0.6 V, at a scan rate of 50 mV s^−1^. The EIS measurements were conducted at a frequency range from 10 kHz to 0.1 Hz at a potential of 0.15 V and amplitude of 5 mV.

### 2.3. Assembly of the Biosensor

**Scheme 1:**
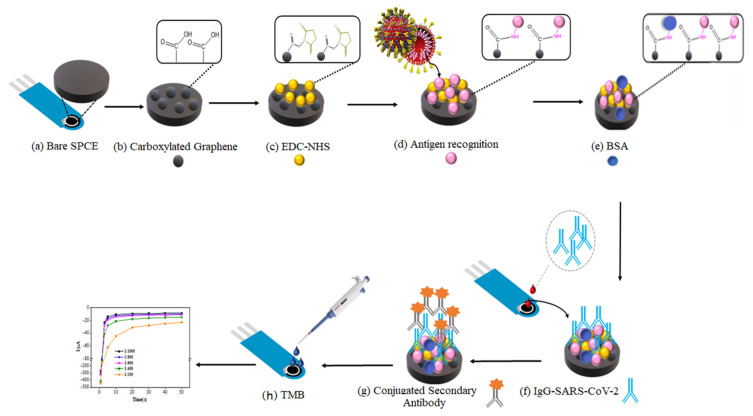
Representation of the immunosensor assembly process and mechanism of indirect detection of analyte target.

The biosensor assembly steps, and the detection of IgG-SARS-CoV-2 antibodies are shown in Scheme 1. Before the electrode modification, the carbon surface was electrochemically activated with five cycles of CV at a potential range between +1 and −1.5 V and a scan rate of 100 mV s^−1^ in a solution of 0.5 mol L^−1^ H_2_SO_4_ [15,16,17]. Then, the surface of the working electrode (WE) of the SPCE was modified with three layers of CG, with 2 mg mL^−1^ of CG solution deposited by drop-casting, and dried at 60 °C, in which the process was repeated 3 times. Subsequently, the -COOH groups of the CG were activated through a carbodiimide reaction with 200 mM EDC and 100 mM NHS for 1 h at room temperature [36,37,38,39,40,41,42]. After washing with PBS buffer, 3.82 μg mL^−1^ of N protein were immobilized on the WE surface for at least 180 min at 4 °C. The biosensor assembly was completed with a blocking step, adding 1% BSA to the WE surface for 60 min at room temperature (Scheme 1b–e). Human serum samples were incubated for 30 min in the WE, followed by the addition of anti-human IgG labeled with horseradish peroxidase (anti-IgG-HRP) (1:1000 *v/v* prepared in PBS solution) for another 30 min at room temperature. Antibody detection was performed by enzymatic reaction using TMB as the substrate for 1 min in the dark. The chronoamperometric technique was used to monitor the current associated with the oxidized TMB reduction process.

### 2.4. Synthesis of Carboxylate Graphene

The CG was prepared by adding 10 mg of graphene to 40 mL of the mixture formed using 30 and 10 mL of concentrated H_2_SO_4_ and HNO_3_, respectively. Then, the mixture was submitted to sonication for 1 h followed by centrifugation for 1 h at 22,000 rpm, with successive wash with Milli-Q water until the supernatant solution achieved pH 7. Then, the synthesized CG was dried using a rotary evaporation system [18,19]. The synthesized CG was characterized by thermogravimetric analysis (TGA), Fourier transform infrared (FTIR), and scanning electron microscopy (SEM). The results can be found in the Supplementary Materials (Figures S1–S3).

### 2.5. Electrochemical Detection

A 7 µL sample of human serum (1:200 *v/v* prepared in PBS solution) was dropped on the WE and incubated for 30 min. After washing with water, 7 µL of horseradish peroxidase-labeled anti-human IgG (anti-IgG-HRP) (1:1000 *v/v* (KPL Inc.) prepared in PBS solution) was added, and incubated for 30 min at room temperature [24,25,43]. Immediately, 100 µL of TMB was placed to promote the enzymatic reaction, and the chronoamperometric (CA) technique followed the reduction process by applying a constant potential of −0.19 V for 50 s (Scheme 1f–h) [44,45]. The analytical signal was based on the absolute value of the current and was recorded at the end of 20 s. For IgG-SARS-CoV-2 detection using the immunosensor, the human serum samples were diluted in PBS from 1:1000 to 1:200, and the response was registered by CA. All measurements were made in triplicate. A new electrode/immunosensor was used for each measurement, which prevented fouling, non-specific adsorption, and reagent leaching. The samples were also analyzed in parallel through the indirect ELISA method, which measured anti-SARS-CoV-2 nucleocapsid immunoglobulin G (IgG) antibody as a reference method, according to LALWANI et al., 2021 [35].

## 3. Results and Discussion

**Figure 1:**
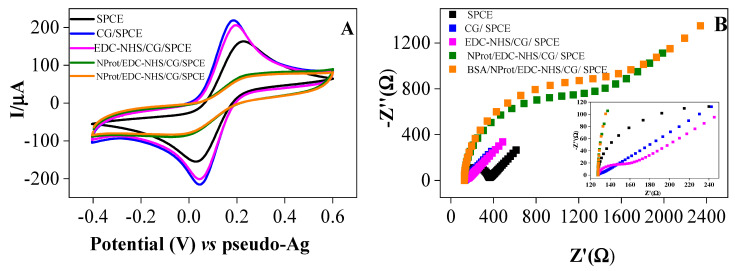
(**A**) Cyclic voltammograms and (**B**) EIS measurements of bare SPCE (black), CG/SPCE (blue), EDC-NHS/CG/SPCE (magenta), N protein/EDC-NHS/CG/SPCE (green), and BSA/N protein/EDC-NHS/CG/SPCE (orange), in 5 mmol L^−1^ [Fe(CN)_6_]^3−/4−^ + 0.1 mol L^−1^ KCl.

Figure 1 shows the CV and EIS results related to the electrochemical characterization of the WE modification steps. Anodic and cathodic peaks can be observed in all electrode modifications, Figure 1A.

In Figure 1A, the redox peaks of bare SPCE exhibited a separation of ~0.2 V and, after the modification with CG, the current peaks from CG/SPCE increased by approximately 30%. Moreover, the potential of anodic and cathodic peaks became closer to each other, showing a decrease in ΔEp of about 0.14 V, suggesting a faster electron transfer between the electrochemical probe and the modified electrode surface that improved the electrochemical performance of SPCE. The modification of the WE with CG increased the capacitive current, which was directly related to the increase in the electroactive area and the roughness factor. The capacitive current is generated due to an accumulation of electrons on the surface of the electrode, increasing the charge of the electrical double layer. Graphene is one of the most used carbon nanomaterials due to its high electrical conductivity and large surface area as compared with carbon nanotubes and graphite. Although graphene can improve the electrical conductivity of composite materials, it also causes very high capacitive currents from SPCEs (>1 µA). To minimize these effects, a potential must be fixed during electrochemical measurements, and the capacitive current contribution is minimized; therefore, the analytical detection was developed using the amperometric technique [15,46,47,48].

The activation of the carboxyl group on the WE surface with EDC-NHS (EDC-NHS/CG/SPCE) promoted a decrease in current peak. A possible explanation is that the insulating feature of these molecules hinders electron transfer from the substrate to the redox couple in the electrolyte interface [49,50]. After the immobilization of antigen (N protein) on the electrode surface, the anodic peak current also decreased significantly to 71 µA. Possibly, the reason is that the immobilized structures act as a electron transfer blocking layer, and it hinders the diffusion of [Fe(CN)_6_]^3−/4−^ redox probe toward the electrode surface. This result proved that the antigen was successfully immobilized on the electrode surface. In the blocking step with BSA (1%), there was a small decrease in current peaks as compared with the previous step. This may be explained by the presence of more biological species on the electrode surface, which can prevent the transfer of electrons. This result was expected since the purpose of BSA was to block excess active groups, avoiding non-specific binding of antibody sites in the detection step.

**Scheme 2:**
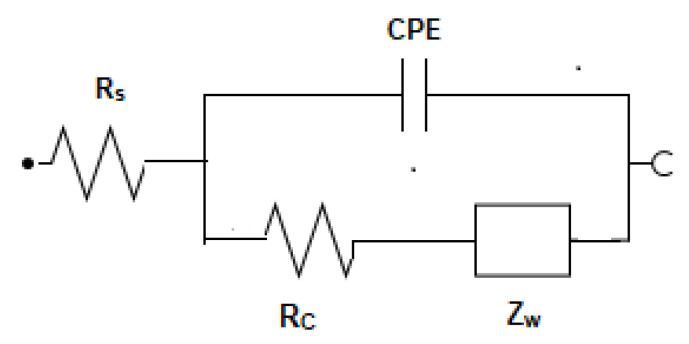
Equivalent circuit for Nyquist plots. R_S_, solution resistance; CPE, constant phase element; R_c_, electron-transfer resistance; Z_W_, Warburg element due to diffusion of the redox couple ([Fe(CN)_6_]^4−^/[Fe(CN)_6_]^3−^) to the interface from the bulk interface from the bulk of the electrolyte.

The EIS analysis (Figure 1B) shows the changes in resistance charge transfer represented in the Nyquist plots. The Nyquist diagrams can be simulated with an equivalent circuit (Scheme 2), where R_S_ is the resistance of the electrolyte solution, CPE is the constant phase element, Rc is the electron-transfer resistance, and Z_W_ is the Warburg element. The semicircle portion from high to intermediate frequencies refers to the kinetic charge transfer process, whereas the straight line at low frequencies arises from the diffusional barrier regarding the redox couple mass transfer [51]. The EIS spectra showed clear differences in signal after integration of each layer onto the SPCE surface starting with the bare SPCE (Rc = 157 Ω), which exhibited a semicircle with a small diameter at high frequencies representative of some SPCE resistance to electron transfer. After modification with CG (Rc = 10 Ω), there was a gradual decrease in resistance to electron transfer, confirmed by the disappearance of the semicircle which was replaced by a straight line indicating a diffusion process. This is due to an increase in conductivity, thus, improving the transfer of electrons between the solution and the surface of the modified electrode. After incubation of the protein (Rc 836 Ω), subsequent blocking with BSA (Rc 1050 Ω) and formation of the immune complex, there was a clear increase in the diameters of the semicircles because of the formation of an insulating layer of protein that covered the surface of the nanomaterials, resulting in an increase in the electron transfer resistance. These changes indicated that each chemical modification on the electrode surface was successfully obtained, corroborating the performance previously observed in cyclic voltammogram.

### 3.1. Optimization of Antigen Immobilization

**Figure 2:**
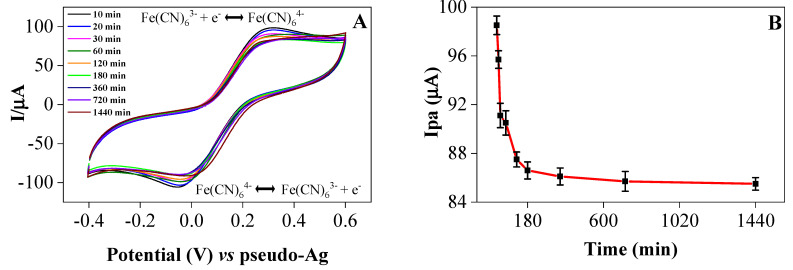
Optimization of N protein immobilization time: (**A**) Cyclic voltammograms in 5 mmol L^−1^ [Fe(CN)_6_]^3−/4−^ + 0.1 mol L^−1^ KCl; (**B**) plots of Ipa (µA) versus tempo (min).

For the immobilization of N protein step, different incubation times (10, 20, 30, 60, 120, 180, 360, 720, and 1440 min) were tested. The electrochemical response was followed by CV and it is illustrated in Figure 2A. The CV peak currents almost stabilized when the incubation time was higher than 180 min. Thus, 180 min was chosen as the optimum immobilization time for the biosensor fabrication Figure 2B.

**Figure 3:**
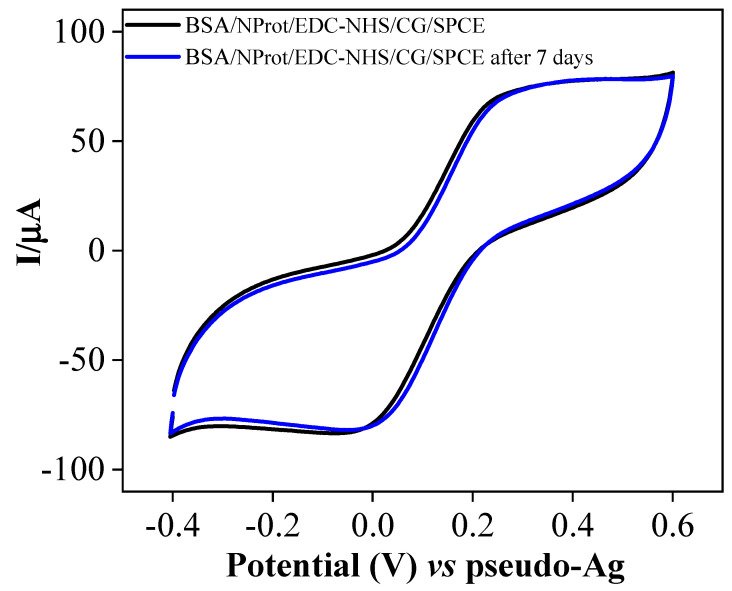
Stability of the immunosensor through cyclic voltammetry in 5 mmol L^−1^ [Fe(CN)6]^3−/4−^ + 0.1 mol L^−1^ KCl.

The stability of the BSA/NProt/EDC-NHS/CG/SPCE immunosensor was evaluated after refrigeration at 4 °C for 7 days. The electrochemical response was followed by CV, and the immunosensor retained approximately 95% of its initial current values (Figure 3). Good long-term stability is expected due to the stability of the CG on the electrode surface and the covalently immobilized Ag on the GC surface.

### 3.2. Cyclic Voltammetric and Chronoamperometric Studies of TMB at SPCEs

**Figure 4:**
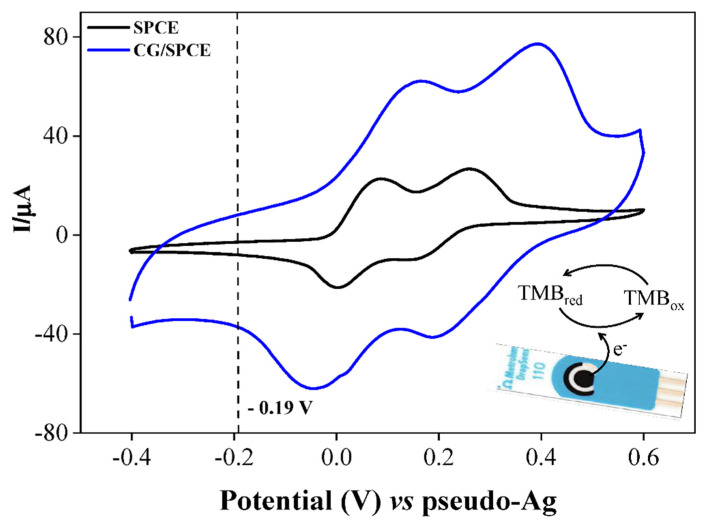
Cyclic voltammograms for SPCE (black) and CG/SPCE (blue) in TMB solution.

CV was used to investigate the oxidation and reduction process of the TMB on a SPCE, by scanning the potential between −400 and 600 mV at a scan rate of 50 mV s^−1^. Figure 4 shows the cyclic voltammograms obtained for bare SPCE and CG/SPCE.

The results showed an increase in peak current in GC/SPCE (Ipa_I_ = 62.2 µA, Ipa_II_ = 76.8 µA, Ipc_I_ = −62.3 µA, and Ipc_II_ = −41.4 µA) as compared with bare SPCE (IpaI = 22.4 µA, IpaII = 26.2 µA, Ipc_I_ = −21.5 µA, and Ipc_II_ = −9.4 µA) due to the high conductivity of the CG composites which contributed to faster electron transfer in the modified electrode. In the immunosensor format, detection of HRP-labeled secondary antibody was based on electrochemical reduction of oxidized TMB by enzymatic reaction. As reported in the literature [23,24,29,30,31,32,33,34,35,36,37,38,39,40], TMB undergoes two-electron oxidation-reduction, which is also characterized in our modified system (CG/SPCE) by two reduction peaks at approximately 195 and −45 mV. Based on these observations, chronoamperometric detection, previously described in the literature [13,24,32,37,41,42], was chosen for the development of this immunosensor. The applied potential of −0.19 V was chosen for the subsequent experiments, since, with this applied potential, the TMB efficiently reduced just the TMB oxidized by the HRP formed on the electrode surface, avoiding an erroneous measurement that could be caused if the applied potential was from the resulting reduction peak [24].

### 3.3. Immunosensor Calibration

**Figure 5:**
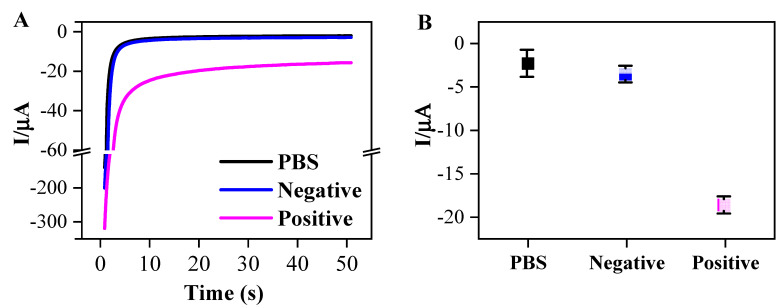
(**A**) Chronoamperograms recorded with PBS, negative and positive controls. Applied potential of −0.19 V vs. pseudo-Ag. (**B**) Current values on the steady-state, negative reference serum, and PBS.

The most suitable electrochemical method to monitor oxidized TMB species was chronoamperometry. The analytical performance of the immunosensor was evaluated in human serum samples diluted in PBS. Under ideal conditions, each sample was incubated separately on the surface of the biosensor device. Furthermore, its repeatability was investigated, in which each sample was tested three times. The chronoamperograms qualitatively discriminated the presence of IgG-SARS-CoV-2 in negative reference serum and positive reference serum to test the viability of the immunosensor. PBS was used as a blank control (Figure 5).

As shown in the I-T curve (Figure 5A), the steady-state signal was reached after 10 s. When IgG-SARS-CoV-2 was present, the steady-state current was approximately −18.72 µA, while for the negative reference serum and PBS it was only −3.56 µA and −2.31 µA, respectively (Figure 5B). From the P/N (ratio of ~5.5), it can be deduced that the immunosensor is capable of distinguishing IgG-SARS-CoV-2 patient serum from healthy human serum.

To test the system’s ability to detect IgG-SARS-CoV-2 in human serum, various concentrations (1:200; 1:400; 1:600; 1:800, and 1:1000) were tested on the immunosensor.

**Figure 6:**
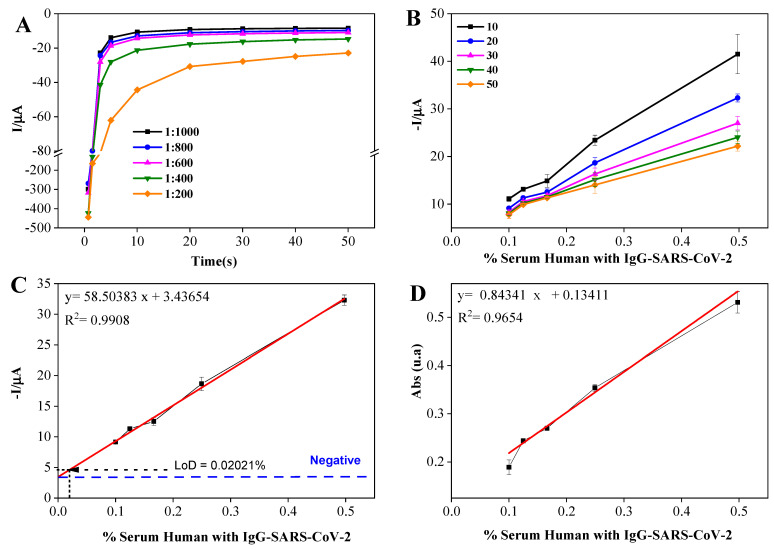
(**A**) Chronoamperometric response measured for different concentrations of human serum with IgG-SARS-CoV-2; (**B**) current values registered from 10 to 50 s for different concentrations of serum human with IgG-SARS-CoV-2; calibration curve for IgG-SARS-CoV-2 determination through (**C**) CA technique and (**D**) ELISA technique. Standard error bars correspond to measurements made on three replicates of each concentration (*n* = 3).

Figure 6A displays chronoamperometric measurements performed at a reduction potential of −0.19 V vs. pseudo-Ag for a total of 50 s. A fast decrease in current can be observed during the first 10 s, caused by electrode polarization. After 10 s, the reaction at the electrode stabilized and began to reach a plateau state. Furthermore, as the human serum dilutions increased, the differences in actual values were smaller.

This is confirmed in Figure 6B, where the current values have been plotted against the concentration of human serum with IgG-SARS-CoV-2 at different reaction times. The results show a linear behavior between the concentration of human serum with IgG-SARS-CoV-2 and the current obtained. However, after 30 s of reaction, linearity is lost as the dilutions of human serum with IgG-SARS-CoV-2 increase, probably due to the low concentration of HRP and consequently of oxidized TMB. The large standard deviations found in high concentrations of human serum with IgG-SARS-CoV-2 can be explained by the electrode passivation effect, as when HRP oxidizes TMB, the resulting blue product is deposited on the electrode surface, blocking it and reducing the current obtained. At high concentrations of labeled antibodies, TMB is oxidized more rapidly, and therefore, the passivation effect is observed earlier [24,43,44].

An indirect electrochemical assay was used to estimate the antibody levels in the serum samples. In Figure 6C, the calibration curves were plotted using absolute values of stable current recorded at time 20 s against the concentration of IgG-SARS-CoV-2 present in the samples. The performance of the immunosensor was evaluated using human serum positive for SARS-CoV-2 concentrations, including 1:1000, 1:800, 1:600, 1:400, and 1:200. It can be observed that the current values increase proportionally with an increase in the concentration of IgG-SARS-CoV-2. The resulting average current values (*n* = 3) were established using the regression equation: y=58.5038 x +3.43654. According to the Miller and Miller method [45], the detection limit of our system was determined taking into account the variability of the chronoamperometric current that was obtained in positive samples (samples without IgG-SARS-CoV-2). The limit of detection (LOD) of our system was determined considering the variability of the chronoamperometric current obtained in the blank samples. The lower LOD was calculated as 3× the standard deviation (SD) blank divided by the slope of the calibration curve. This limit was established as a current value of 4.59 µA corresponding to a % concentration of IgG-SARS-CoV-2 of 0,02021%, which is equivalent to a dilution factor of 1:4954. A linear graph is obtained with R^2^ of 0.9908, indicating that the time at 20 s is suitable for this study. The resolution of the curve fitting indicated a sensitivity (expressed as the slope of the I vs. % human serum with IgG-SARS-CoV-2) of 3.43654/% human serum with IgG-SARS-CoV-2 in the range from 1:1000 to 1:200 *v*/*v*. As shown, the fabricated biosensor can be used for the detection of IgG-SARS-CoV-2 in real human serum sample.

To confirm the results obtained by CA for the detection of IgG-SARS-CoV-2, experiments through ELISA technique were performed. The calibration curve (Figure 6D) was obtained by plotting absorbance versus % human serum with IgG-SARS-CoV-2. The obtained results show a linear behavior in the concentration range from 1:1000 to 1:200, with a calculated LOD of 1:2023 (0.04940%). The regression equation is defined as y=0.84341 x +0.13411 , com R^2^ = 0.9654.

For the feasibility of the biosensor device in the detection of IgG-SARS-CoV-2 in biological samples, 10 clinical samples were also tested to verify the biosensor’s viability in detecting IgG-SARS-CoV-2 antibody in biological samples and the results obtained are summarized in Table 1 and compared with the ELISA test. The results show a possible application of the biosensor to detect IgG-SARS-CoV-2 in blood serum samples.

## 4. Conclusions

Effective monitoring and surveillance of COVID-19 in primary health care is essential to prevent the emergence of outbreaks. In this work, a highly sensitive electrochemical immunosensor for IgG-SARS-CoV-2 nucleocapsid protein detection was successfully developed. Modification of an SPCE with CG as the sensor platform and implementation of an indirect immunoassay format was successfully used for the detection of IgG-SARS-CoV-2 nucleocapsid protein. The electrochemical study revealed the immobilization of Ag in CG/SPCE, the CG aimed to improve the electrochemical signal and the selectivity in the immobilization of the N protein, indicating the suitability of this sensor platform to be developed as a biosensor. The low LOD (0.02021%) allows detecting smaller amounts of IgG-SARS-CoV-2 in real samples. In addition, the concentration and immunodetection steps can be effectively integrated into a single support, allowing for a more direct and faster system that can provide results in approximately 1 h. Thus, the detection system developed can overcome one of the most significant disadvantages of the ELISA method, i.e., the detection time. As a result, we achieved our goal of a fast, cost-effective, stable, and easy-to-use system for detecting IgG-SARS-CoV-2.

## Figures and Tables

**Table 1:** Detection of IgG-SARS-CoV-2 in blood serum samples using the proposed immunosensor and the ELISA test.

Patients	Immunosensor	ELISA
1	+	+
2	+	+
3	+	+
4	+	+
5	+	+
6	−	−
7	−	−
8	−	−
9	−	−
10	−	−

## Data Availability

The data presented in this study are available in DOI: 10.1016/j.ijid.2021.07.017.

## Supplementary Materials

The following supporting information can be downloaded at https://www.mdpi.com/article/10.3390/bios12121161/s1, Figure S1: TG curves of decomposition of graphene and carboxylated graphene, Figure S2: ATR-FTIR spectra of graphene and carboxylated graphene, Figure S3: Scanning electron microscope (SEM) images of (A) bare SPCE, (B) graphene/SPCE, and (C) CG/SPCE. [39,40,52,53,54,55,56,57,58,59,60].
