# Supplementary material for: An Electrochemical Immunosensor Based on Carboxylated Graphene/SPCE for IgG-SARS-CoV-2 Nucleocapsid Determination
Source: Biosensors (Basel). 2022 Dec 13;12(12):1161. doi: 10.3390/bios12121161 (PMC9775996; doi:10.3390/bios12121161)
Supplement: Supplementary file 1 [file biosensors-12-01161-s001.zip › biosensors-1969746-supplementary.pdf]

biosensors

Supplementary Information

An Electrochemical Immunosensor Based on Carboxylated Graphene/SPCE for IgG-SARS-CoV-2 Nucleocapsid Determination

Luciana de Souza Freire, Camila Macena Ruzo, Barbara Batista Salgado, Ariamna María Dip Gandarilla, Yonny Romaguera-Barcelay, Ana P. M. Tavares, Maria Goreti Ferreira Sales, Isabelle Cordeiro, Jaila Dias Borges Lalwani, Robert Matos, Henrique Fonseca Filho, Spartaco Astofi-Filho, Ştefan Ţălu, Pritesh Lalwani and Walter Ricardo Brito

Characterization of Carboxylated Graphene

The carboxylated graphene was characterized using Thermal Gravimetric Analysis (TGA) (Shimadzu TGA-50H, nitrogen atmosphere, flow rate of 50 mL/min, platinum cell, at 10 °C/ min). **Error! Reference source not found.** shows the comparative TGA of graphene and carboxylated graphene. The decomposition process includes three main steps: (i) solvent and water evaporation, (ii) decomposition of oxygenated functional groups, (iii) CO formation. In the evaporation step at 0 °C to 170 °C, a weight loss of ~3% and 14% was archived for graphene and carboxylated graphene, respectively. These weight losses are associated to the evaporation of solvent and constitutional water and the adsorption of gases on the material surface during the experiment [1–3]. The next stage registered a major weight loss (48%) for the carboxylic group of graphene, from 170 °C to 610 °C. The decomposition of the organic materials in carbonates occurs in this temperature range. Organic cation carbonization can be considered as the major competition reaction during the organic material decomposition due to the oxidation of the organic compounds in air. In the third stage, minor weight loss of approximately 1% for graphene and 14% for carboxylated graphene takes place in the 610 °C – 800 °C temperature range, which can be associated with the decomposition of CO species [3–7].

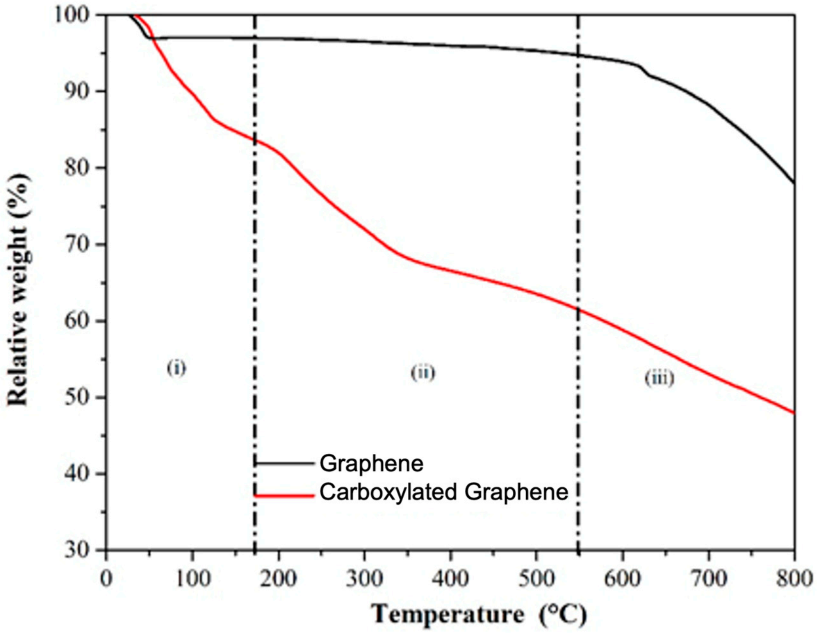

Figure S1. TG curves of decomposition of graphene and carboxylated graphene.

The carboxylation of graphene was observed by ATR-FTIR (MIRacle with a ZnSe Crystal Plate Pike®), which was installed on a Nicolet® 6700 FT-IR spectrometer equipped with a cooled MCT detector with N_2 liquid (**Error! Reference source not found.**). It was characterized by a band centered at 1718 cm^{-1}, 1573 cm^{-1} and 1240 cm^{-1}, corresponding to the C=O stretching vibration of carboxylic groups, Ring str. of hexagonal structure and HOC bend of hydroxyl groups [7,8].

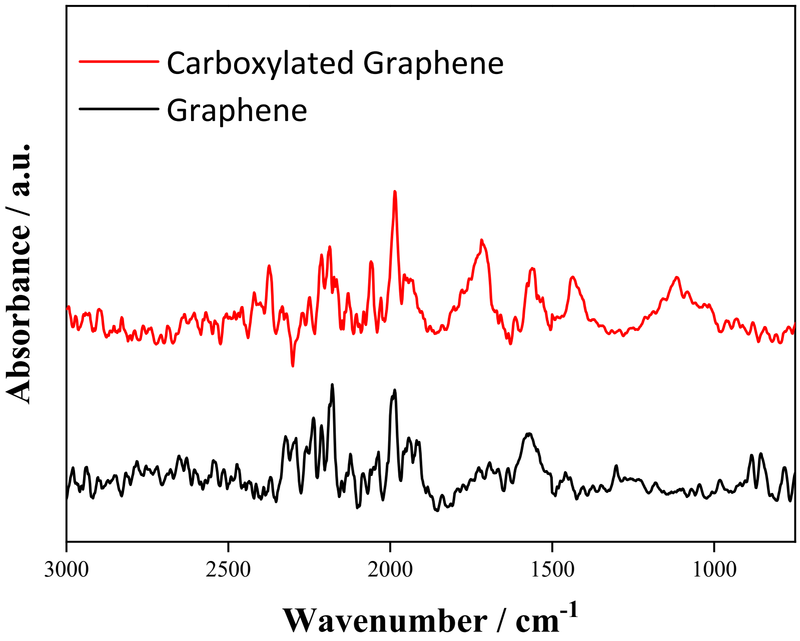

Figure S2. ATR-FTIR spectra of graphene and carboxylated graphene.

The surface morphology of the bare SPCE, Graphene/SPCE and CG/SPCE was analyzed using scanning electron microscopy (SEM, Tescan, model Vega 3) operated with an accelerating voltage of 15 KV (INCA energy, United Kingdom). Prior to the SEM imaging, the surface of the SPCE was coated with a thick gold film (5 nm), and the images collected are represented as **Error! Reference source not found.**. The bare electrode had a high surface roughness and flake-like irregular microstructures and cracks. Unlike the bare electrode, the graphene/SPCE surface showed nanoplates, and there were some wrinkles and folds on the surface of the graphene nanoplates. The CG/SPCE surface was more uniform due to the good surface coverage of CG. Compared to Graphene/SPCE, the general structure of CG was greatly changed, and the thin plates were well exfoliated [9–11].

Overall, the chemical and physical characterization performed on graphene confirmed the presence of carboxyl group on its surface. Moreover, drop casting proved an efficient method to deposit CG on the working surface of SPCE.

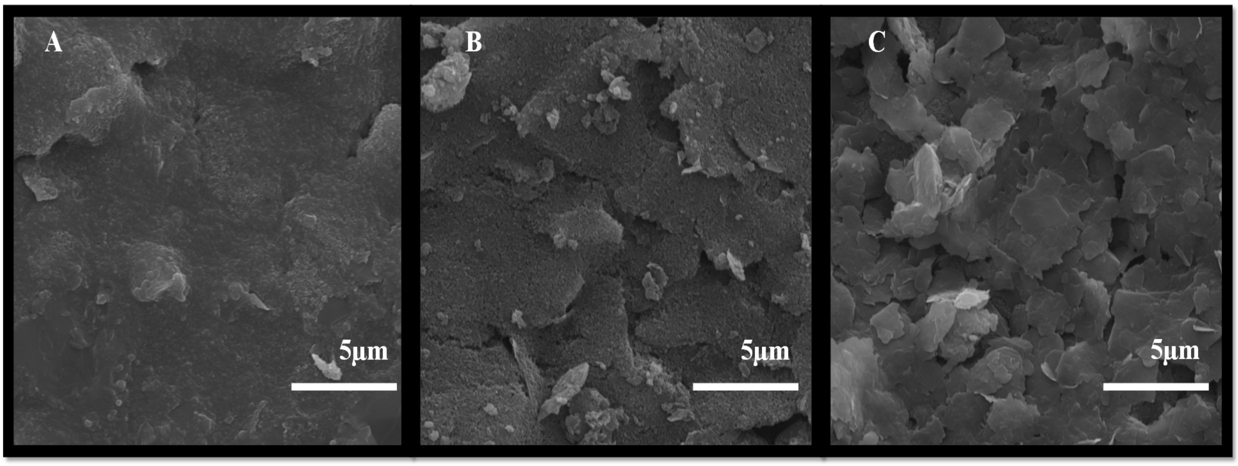

Figure S3. Scanning electron microscope (SEM) images of (A) bare SPCE, (B) graphene/SPCE and (C) CG/SPCE.

References

1. Verdoodt, N.; Basso, C.R.; Rossi, B.F.; Pedrosa, V.A. Development of a rapid and sensitive immunosensor for the detection of bacteria. *Food Chem.* **2017**, *221*, 1792–1796. https://doi.org/10.1016/j.foodchem.2016.10.102.
2. Jaiswal, N.; Pandey, C.M.; Soni, A.; Tiwari, I.; Rosillo-Lopez, M.; Salzmann, C.G.; Malhotra, B.D.; Sumana, G. Electrochemical genosensor based on carboxylated graphene for detection of water-borne pathogen. *Sens. Actuators B Chem.* **2018**, *275*, 312–321. https://doi.org/10.1016/j.snb.2018.07.055.
3. Chhabra, V.A.; Deep, A.; Kaur, R.; Kumar, R. Functionalization of Graphene using Carboxylation Process, *Int. J. Sci. Emerg. Technol. with Latest Trends*, **2012**, *4*, 13–19.
4. Eigler, S.; Dotzer, C.; Hirsch, A.; Enzelberger, M.; Müller, P. Formation and decomposition of CO2 intercalated graphene oxide, *Chem. Mater.*, **2012**, *24*, 1276–1282, DOI: 10.1021/cm203223z.
5. Chhabra, V.; Singh, M.; Deep, A. Carboxylated Graphene-Indium Tin Oxide Hetrojunction for Bio-Sensing, *Nanotechnol. Nov. Perspect. Prospect.*, **2015**, *1*, 414–410, DOI: 10.13140/RG.2.1.1998.6806.
6. Kuila, T.; Bose, S.; Mishra, A.K.; Khanra, P.; Kim, N.H.; Lee, J.H. Chemical functionalization of graphene and its applications, *Prog. Mater. Sci.*, **2012**, *57*, 1061–1105, DOI: 10.1016/j.pmatsci.2012.03.002.
7. Naebe, M.; Wang, J.; Amini, A.; Khayyam, H.; Hameed, N.; Li, L.H.; Chen, Y.; Fox, B. Mechanical Property and Structure of Covalent Functionalised Graphene/Epoxy Nanocomposites, *Sci. Rep.*, **2014**, *4*, 1–7, DOI: 10.1038/srep04375.
8. Vasile, E.; Pandele, A.M.; Andronescu, C.; Selaru, A.; Dinescu, S.; Costache, M.; Hanganu, A.; Raicopol, M.D.; Teodorescu, M. Hema-Functionalized Graphene Oxide: a Versatile Nanofiller for Poly(Propylene Fumarate)-Based Hybrid Materials, *Sci. Rep.*, **2019**, *9*, 18685, DOI: 10.1038/s41598-019-55081-2.
9. Shao, G.; Liu, P.; Zhang, K.; Li, W.; Chen, X.; Ma, F. Mechanical properties of graphene nanoplates reinforced copper matrix composites prepared by electrostatic self-assembly and spark plasma sintering, *Mater. Sci. Eng. A*, **2019**, *739*, 329–334, DOI: 10.1016/j.msea.2018.10.067.
10. Park, K.W.; Jung, J.H. Spectroscopic and electrochemical characteristics of a carboxylated graphene-ZnO composites, *J. Power Sources*, **2012**, *199*, 379–385, DOI: 10.1016/j.jpowsour.2011.10.016.
11. Gu, Y.; Ju, C.; Li, Y.; Shang, Z.; Wu, Y.; Jia, Y.; Niu, Y. Detection of circulating tumor cells in prostate cancer based on carboxylated graphene oxide modified light addressable potentiometric sensor, *Biosens. Bioelectron.*, **2015**, *66*, 24–31, DOI: 10.1016/j.bios.2014.10.070.
